# Feasibility of tumor-informed circulating tumor DNA for detecting minimal residual disease in surgically resected biliary tract cancer

**DOI:** 10.1371/journal.pone.0341432

**Published:** 2026-01-21

**Authors:** Younghee Park, Kyung Su Kim, Hyunji Jo, Hwang-Phil Kim, Dong Soo Kyung, Youngeun Yoo, Seog Ki Min, Eun Mi Nam, Kyubo Kim

**Affiliations:** 1 Department of Radiation Oncology, Ewha Womans University College of Medicine, Seoul, Korea; 2 Department of Radiation Oncology, Seoul National University Hospital, Seoul National University College of Medicine, Seoul, Korea; 3 Division of Hematology-Oncology, Department of Internal Medicine, Ewha Womans University College of Medicine, Seoul, Korea; 4 Clinical Research, IMBdx, Seoul, Korea; 5 Department of Pathology, Ewha Womans University College of Medicine, Seoul, Korea; 6 Department of Surgery, Ewha Womans University College of Medicine, Seoul, Korea; 7 Department of Radiation Oncology, Seoul National University Bundang Hospital, Seoul National University College of Medicine, Seongnam, Korea; School of Pharmacy - Bandung Institute of Technology, INDONESIA

## Abstract

**Background:**

We prospectively investigated the potential of tumor-informed circulating tumor DNA (ctDNA) for detecting minimal residual disease (MRD) in surgically resected biliary tract cancer (BTC).

**Methods:**

Personalized panels were developed using individual variants identified from the whole-exome sequencing of surgical specimens from each patient. Two sequential blood samples, collected preoperatively and within 6 weeks after surgery, were analyzed. A positive ctDNA result was defined as the identification of two or more patient-specific mutations.

**Results:**

A total of 18 patients were enrolled. However, 1 patient was excluded due to inoperability detected during surgery, and personalized target panels could not be created for 3 patients due to a low number of target variants, resulting in the analysis of 14 patients. There was a tendency for a higher preoperative ctDNA positivity rate in an advanced overall stage (100% in stage III-IV vs. 44.4% in stage I-II, p = 0.126) and node-positive disease (100% in node-positive vs. 60% in node-negative, p = 0.210). After a median follow-up of 17.4 months (range, 11.4–31.1), the 1-year and 2-year progression-free survival (PFS) rates were 78.6% and 58.2%, respectively. Changes in ctDNA positivity status were negative to negative in 3 patients (25.0%), positive to negative in 3 patients (25.0%), and positive to positive in 6 patients (50%). Additionally, there was a trend for an association between poorer PFS and both preoperative and postoperative ctDNA positivity. Among ctDNA-negative patients before surgery, no progression was observed; however, 5 out of 10 ctDNA-positive patients experienced progression. Postoperatively, only 1 out of 6 ctDNA-negative patients experienced progression, in contrast to 3 out of 6 patients with a positive ctDNA result.

**Conclusions:**

Although limited by the small sample size, our results may indicate a possible role for tumor-informed ctDNA analysis in detecting MRD in surgically resected BTC and warrant further validation in larger studies.

## Introduction

Biliary tract cancer (BTC), including intrahepatic cholangiocarcinoma, extrahepatic cholangiocarcinoma, and gallbladder cancer, accounts for 3% of all gastrointestinal malignancies. Although it is a relatively rare malignancy, its incidence and mortality rates have been increasing [1]. Surgical resection is the primary treatment and only curative option; however, only 20–30% of patients are eligible because of late detection in advanced stages [2].

After curative resection for early-stage resectable BTC, approximately 60–70% of patients experience recurrent disease with poor outcomes. Therefore, extensive research has been conducted on adjuvant treatment including radiotherapy and/or systemic therapy. Currently, standard treatment includes 6 months of adjuvant capecitabine, as supported by findings from the BILCAP study [3]. This study demonstrated the overall survival (OS) benefit of adjuvant capecitabine over observation for resected BTC. However, the 5-year relapse-free survival (RFS) rate was only 34% for the capecitabine group and 50% for all patients with local recurrence. Therefore, there is an urgent need to improve treatment strategies for the majority of patients who will experience recurrence.

Circulating tumor DNA (ctDNA) from minimal residual disease (MRD) has shown prognostic value in various solid cancers [4,5]. Although residual ctDNA is known to be associated with poor disease-free survival (DFS) in colorectal cancer [4], recent studies have focused on the potential of ctDNA as a guide to the escalation or de-escalation of adjuvant therapy and early detection of recurrence [6,7]. However, there are limited clinical studies related to MRD detection using ctDNA in BTC [8–10].

Here, we aimed to explore the potential utility of MRD detection using ctDNA in resected BTC to establish more definitive evidence and improve patient outcomes through personalized adjuvant treatment strategies.

## Materials and methods

### Patients and sample collection

The study protocol was approved by the Institutional Review Board of Ewha Womans University Seoul Hospital (approval number: 2021-01-019) and all patients provided written informed consent. Patients diagnosed with BTC and scheduled for radical surgery between May 2021 and May 2023 were prospectively enrolled in this study. Patients who received neoadjuvant treatment before surgery were excluded. Blood samples were collected according to the study protocol. Specifically, blood sample was obtained prior to surgery and within 6 weeks post-surgery, before any adjuvant therapy started. For patients who underwent adjuvant therapy, additional samples were collected at the time of treatment response assessment, whereas for those who did not receive adjuvant therapy, samples were obtained every 3 months during routine follow-up visits. Formalin-fixed, paraffin-embedded (FFPE) tissues of surgical specimens were obtained.

### DNA sequencing and analysis

ctDNA sequencing and analysis were performed as previously described [11], and Genomic DNA was extracted from FFPE surgical specimens using the Maxwell® RSC DNA FFPE Kit (Promega, Madison, WI, USA) and from peripheral blood mononuclear cells using the Maxwell® Blood DNA Kit (Promega, Madison, WI, USA) following the manufacturer’s instructions. The Illumina-compatible whole-exome sequencing (WES) library was prepared and sequenced using the NovaSeq 6000 Sequencing System (Illumina, San Diego, CA, USA).

Cell-free DNA (cfDNA) was extracted using the Maxwell^®^ RSC cfDNA Plasma Kit (Promega, Madison, WI, USA) and quantified by Cell-free DNA ScreenTape Analysis with the 4200 TapeStation System (Agilent, Santa Clara, CA, USA). The Illumina-compatible library was prepared using a bespoke panel and sequenced using the NovaSeq 6000 Sequencing System (Illumina, San Diego, CA, USA).

For the analysis of sequencing data, adapter sequences and low-quality reads were trimmed using fastp [12]. The trimmed reads were aligned to the reference genome (hg38) using bwa [13] and then further processed following the GATK Best Practices recommendations. Somatic variants were identified using Mutect2 software with the tumor normal paired option [14]. Likely false positives were removed. Variants were annotated using several databases: CancerHotspot [15], ClinVar [16], dbSNP [17], gnomAD [18], and Korean Germline DB [19]. Among the annotated variants, up to 100 variants were further selected to design a bespoke panel (BSP) using the proprietary selection algorithm of IMBdx (Seoul, Korea).

For the analysis of sequencing data, raw FASTQ files were trimmed using fastp software. Unique molecular identifiers were extracted from the sequencing data and used to generate high-quality sequences. Variants were identified using VarDict software targeting the selected variant loci. Variants with a low quality or low depth were filtered. ctDNA was defined as positive if two or more variants were detected, based on findings from previous study using the same assay platform [11].

### Statistical analysis

Progression was defined as the development of new lesions or the radiologic progression of pre-existing lesions on follow-up imaging. Changes in tumor markers (CA19−9 or CEA) were also considered in conjunction with imaging findings, but an isolated elevation of tumor markers was not regarded as recurrence. Progression-free survival (PFS) was calculated from the date of the surgical resection to the detection of disease progression or last follow-up. The actuarial survival rate was calculated with the Kaplan-Meier method, and differences were verified with the log-rank test. Pearson’s Chi-square test was used to verify differences in categorical variables. All statistical analyses were performed using SPSS software version 27.0 (IBM Corp., Armonk, NY, USA).

## Results

### Patient and tumor characteristics

**Fig 1:**
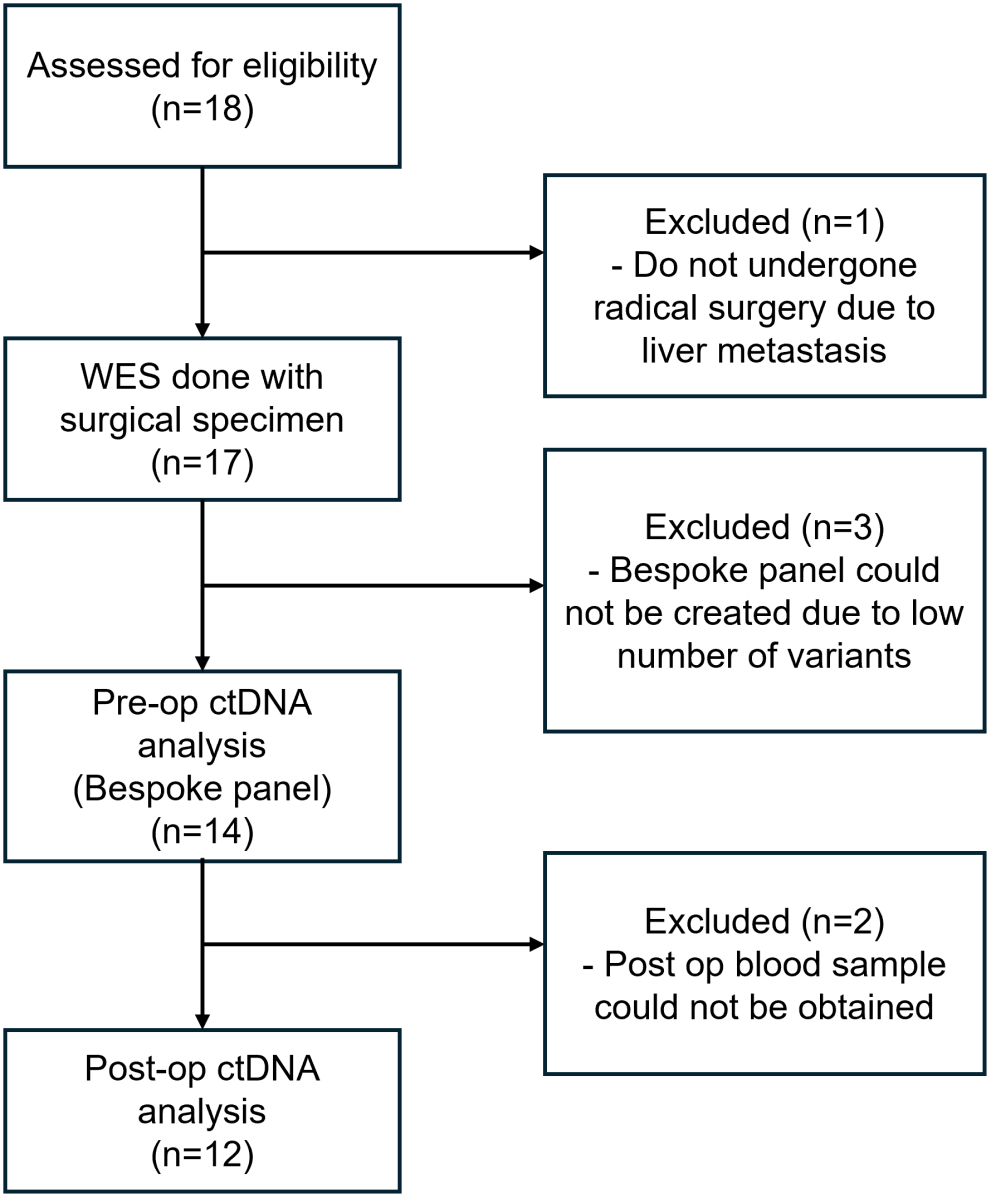
Patient enrollment and exclusion flow chart.

A total of 18 patients were enrolled in this study. However, 1 patient who did not complete the planned radical surgery was excluded, and personalized target panels could not be created for 3 patients due to a low number of target variants. Two patients were clinically diagnosed with distal common bile duct cancer but later confirmed as pancreatic origin after surgery: one with poorly differentiated adenocarcinoma (pT2N1) and the other with moderately differentiated adenocarcinoma (pT2N0). The third had a moderately differentiated adenocarcinoma of the distal common bile duct (pT1N0). Therefore, the preoperative ctDNA of 14 patients was analyzed. Among them, we could not obtain postoperative samples from 2 patients; thus, the postoperative ctDNA of 12 patients was analyzed (Fig 1). The detailed patient and tumor characteristics are presented in Table 1. The median age was 68.5 years (range, 56–81), and 8 patients (57.1%) were male. The specific tumor subsites were as follows: intrahepatic bile duct in 3 patients (21.4%), perihilar in 2 patients (14.3%), distal in 4 patients (28.6%), gallbladder in 3 patients (21.4%), and both gallbladder and distal bile duct in 2 patients (14.3%). A total of 9 patients (64.3%) had stage I or II tumors, and 4 patients (28.6%) had node metastases. The resection margin was negative in 10 patients (71.4%), and 5 patients (35.7%) received adjuvant therapy.

**Table 1:** Patient and tumor characteristics (n = 14).

Characteristics		n	%
Gender	Male	8	57.1
	Female	6	42.9
Age (years)	Median	68.5	
	Range	56-81	
Tumor location	Intrahepatic	3	21.4
	Perihilar	2	14.3
	Distal	4	28.6
	GB	3	21.4
	GB + distal	2	14.3
Stage	I	2	14.3
	II	7	50.0
	III	4	28.6
	IV	1	7.1
T stage	T1	2	14.3
	T2	10	71.4
	T3	1	7.1
	T4	1	7.1
Node metastasis	No	10	71.4
	Yes	4	28.6
Resection margin	Negative	10	71.4
	Involved by invasive carcinoma	3	21.4
	Involved by high grade dysplasia	1	7.1
Adjuvant treatment	No	9	64.3
	Yes	5	35.7

Abbreviations: GB, gallbladder.

### Genomic profile of tissue and ctDNA

The median sequencing depths for surgical specimen, preoperative ctDNA, and postoperative ctDNA were 369 (range, 119–663), 194,940 (range, 129,184–241,052), and 192,544 (range, 124,252–236,019), respectively (Table 2).

**Table 2:** Sequencing summary.

Sample type	Median sequencing depth (range)
Tumor (n = 17)	369 (119-663)
PBMC (n = 17)	108 (78-159)
cfDNA (preoperative) (n = 14)	194,940 (129,184-241,052)
cfDNA (postoperative) (n = 12)	192,544 (124,252−236,019)

Abbreviations: PBMC; peripheral blood mononuclear cells. cfDNA, cell-free DNA.

It is crucial to include large-scale mutations for a sensitive tumor-informed MRD test result [11,20,21]. Additionally, the cancer type of the current study cohort is known to have an intermediate number of mutations [22]. Based on this information, 3 patients with fewer than 10 identifiable mutations were excluded from the panel design.

**Fig 2:**
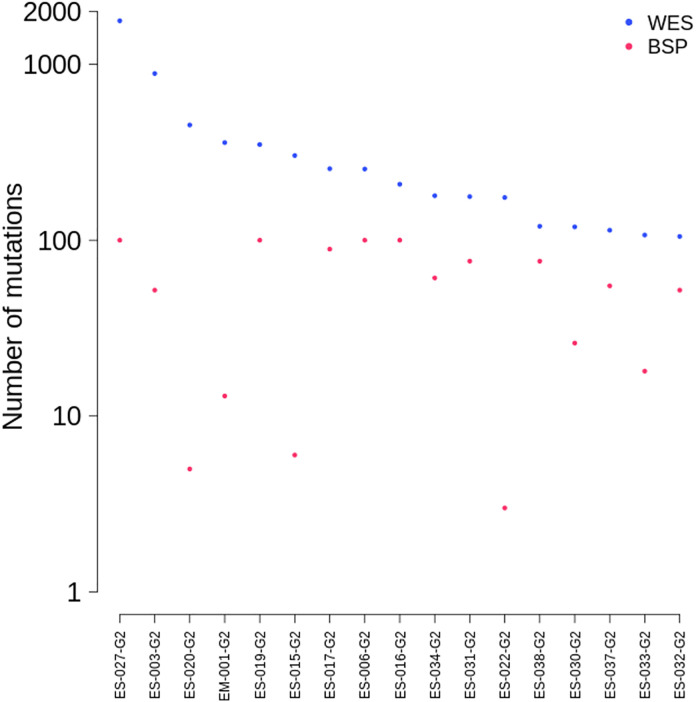
The number of mutations discovered from whole exome sequencing and selected for bespoke panel design. X-axis was sorted by the number of mutations discovered in whole exome sequencing and the log-scale transformation was applied to y-axis. WES: Mutations discovered from whole exome sequencing, BSP: Mutations considered as suitable for bespoke panel design.

**Fig 3:**
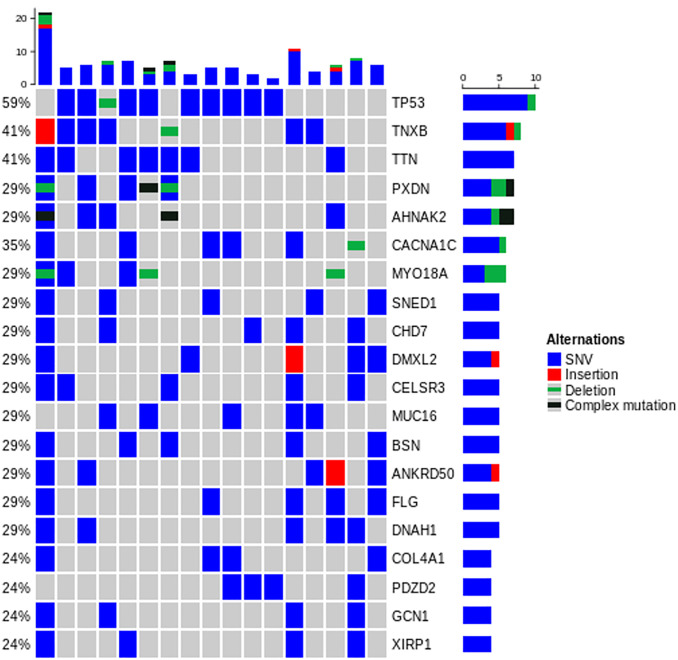
Mutational landscape of whole exome sequencing (top 20 displayed).

**Fig 4:**
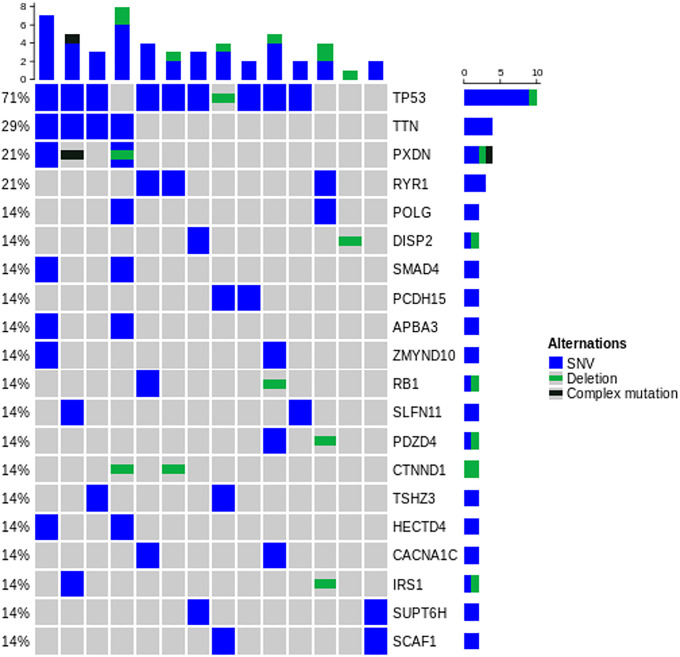
Mutational landscape for bespoke panel design (top 20 displayed).

**Fig 5:**
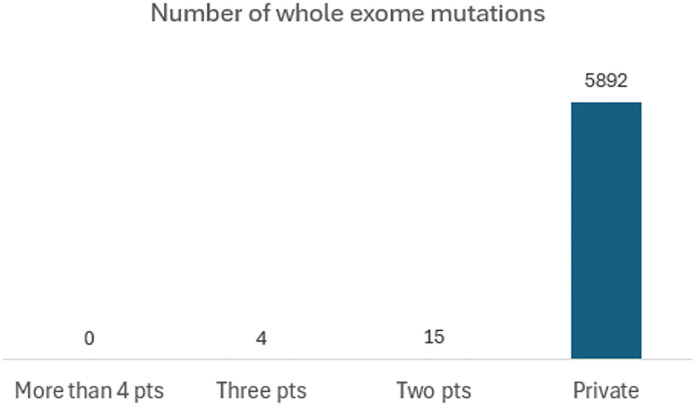
Distribution of mutations from surgical specimen. The number above the bar indicates the number of mutations belongs to the group listed below. Private: Mutation discovered only one patient. Two pts: Mutation discovered across two patients. Three pts: Mutation discovered across three patients. More than 4 pts: Mutation discovered across greater than or equal to the 4 patients.

Based on WES, the median numbers of mutations were 208 for surgical specimen (range, 105–1,770) and 68 for BSP (range, 13–100) (Table 3 and Fig 2). The detailed molecular characteristics are presented in Figs 3 and 4. The majority of mutations (99.7%, 5892/6011) identified in surgical specimen from BTC patients were private mutations, which were found in only 1 patient (Fig 5).

**Table 3:** The number of mutations discovered from whole exome sequencing and selected for bespoke panel design.

Mutation type	Median number of mutations (range)
WES discovered (N = 17)	208 (105−1,770)
BSP selected (N = 14)	68 (13-100)

Abbreviations: WES, whole exome sequencing; BSP, bespoke panel.

### ctDNA positivity and changes

Before surgery, 10 out of 14 patients (71.4%) showed ctDNA positivity. After surgery, 6 out of 12 patients (50.0%) showed ctDNA positivity. Changes in ctDNA positivity status were as follows: negative to negative in 3 patients (25.0%), positive to negative in 3 patients (25.0%), and positive to positive in 6 patients (50.0%).

**Fig 6:**
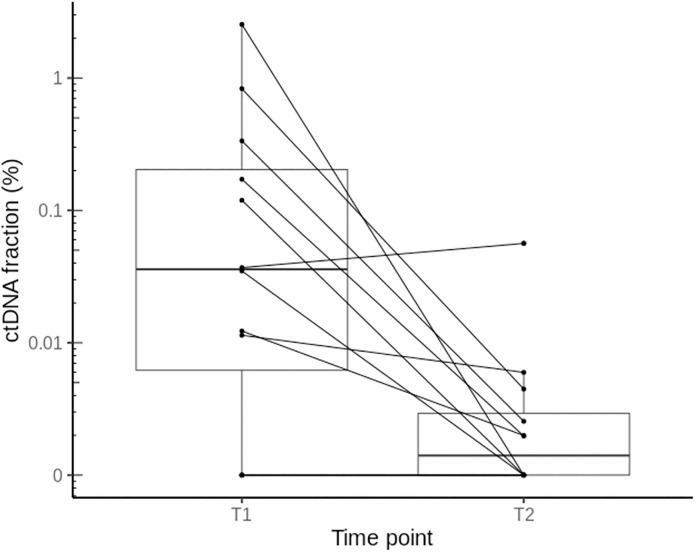
The boxplot of ctDNA fraction at preoperative and postoperative time points. Each pair of samples are connected by the solid line. The ctDNA fraction indicates the total amount of ctDNA molecules detected divided by the total cell-free DNA sequenced. Y-axis scale is log10 transformed. T1: preoperative, T2: postoperative.

Among patients with both preoperative and postoperative ctDNA results, the median numbers of detected mutations in preoperative and postoperative ctDNA were 25 (range, 0–93) and 2 (range, 0–65), respectively, and the corresponding ctDNA fractions were 0.023 (range, 0–2.5) and 0.0005 (range, 0–0.06), respectively (Table 4 and Fig 6).

**Table 4:** The number of detected mutations and ctDNA fraction in patients' plasma.

Time point	Median number of mutations (range)	Median ctDNA fraction (%, range)
Preoperative(n = 14)	25 (0-93)	0.035 (0-2.5)
Postoperative (n = 12)	2 (0-65)	0.0005 (0-0.06)

Abbreviations: ctDNA, circulating tumor DNA.

### ctDNA positivity status and patient and tumor characteristics

The differences in patient and tumor characteristics according to ctDNA positivity are summarized in Table 5. A higher preoperative ctDNA positivity rate was noted among patients with an advanced overall stage (100% in stage III-IV vs. 55.6% in stage I-II, p = 0.221); however, this difference was not statistically significant. A similar trend was observed for node metastasis, with 100.0% preoperative ctDNA positivity among patients with node-positive disease compared to 60.0% among those with node-negative disease (p = 0.251). For postoperative ctDNA, the positivity rate was higher among patients with node-positive disease (75.0%) compared to those with node-negative disease (37.5%, p = 0.545).

**Table 5:** ctDNA positivity and patient and tumor characteristics.

Characteristics		Preoperative ctDNA (n = 14)				Postoperative ctDNA (n = 12)			
		n	Negative	Positive	*p*-value	n	Negative	Positive	*p*-value
Gender	Male	8	3 (37.5)	5 (62.5)	0.580	7	3 (42.9)	4 (57.1)	1.000
	Female	6	1 (16.7)	5 (83.3)		5	3 (60)	2 (40)	
Tumor location	Intrahepatic	3	0 (0.0)	3 (100.0)	0.133	3	1 (33.3)	2 (66.7)	0.119
	Perihilar	2	0 (0.0)	2 (100.0)		2	1 (50.0)	1 (50.0)	
	Distal	4	3 (75.0)	1 (25.0)		4	4 (100.0)	0 (0.0)	
	GB	3	1 (33.3)	2 (66.7)		2	0 (0.0)	2 (100.0)	
	GB & distal	2	0 (0.0)	2 (100.0)		1	0 (0.0)	1 (100.0)	
Stage	I – II	9	4 (44.4)	5 (55.6)	0.221	7	4 (57.1)	3 (42.9)	1.000
	III – IV	5	0 (0.0)	5 (100.0)		5	2 (40.0)	3 (60.0)	
T stage	T1-T2	12	4 (33.3)	8 (66.7)	1.000	10	5 (50.0)	5 (50.0)	1.000
	T3-T4	2	0 (0.0)	2 (100.0)		2	1 (50.0)	1 (50.0)	
Node metastasis	No	10	4 (40.0)	6 (60.0)	0.251	8	5 (62.5)	3 (37.5)	0.545
	Yes	4	0 (0.0)	4 (100.0)		4	1 (25.0)	3 (75.0)	
Resection margin	Negative	10	3 (30.0)	7 (70.0)	1.000	8	4 (50.0)	4 (50.0)	1.000
	Positive	4	1 (25.0)	3 (75.0)		4	2 (50.0)	2 (50.0)	
Adjuvant treatment	No	9	3 (33.3)	6 (66.7)	1.000	7	4 (57.1)	3 (42.9)	1.000
	Yes	5	1 (20.0)	4 (80.0)		5	2 (40.0)	3 (60.0)	

Abbreviations: GB, gallbladder; ctDNA, circulating tumor DNA.

### ctDNA positivity and prognosis of patients

**Fig 7:**
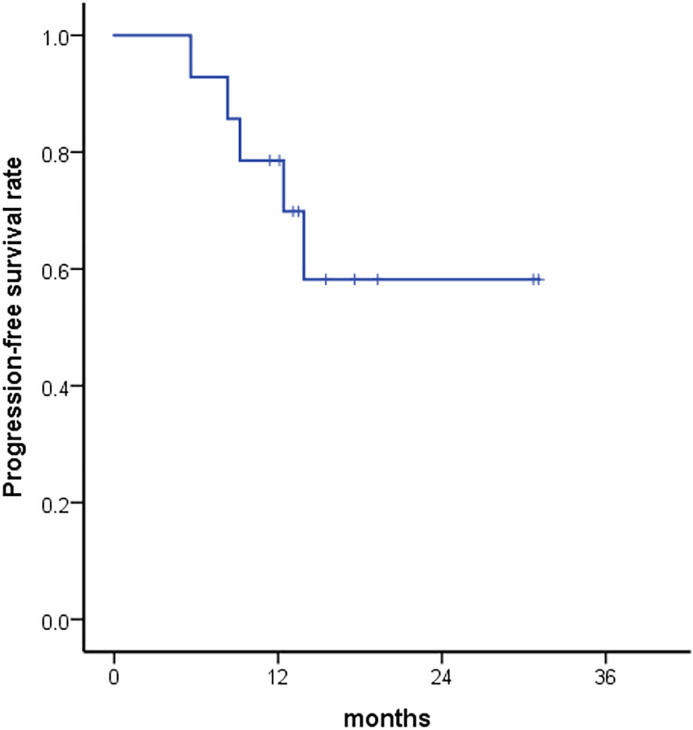
Kaplan-Meier curve for progression-free survival.

**Fig 8:**
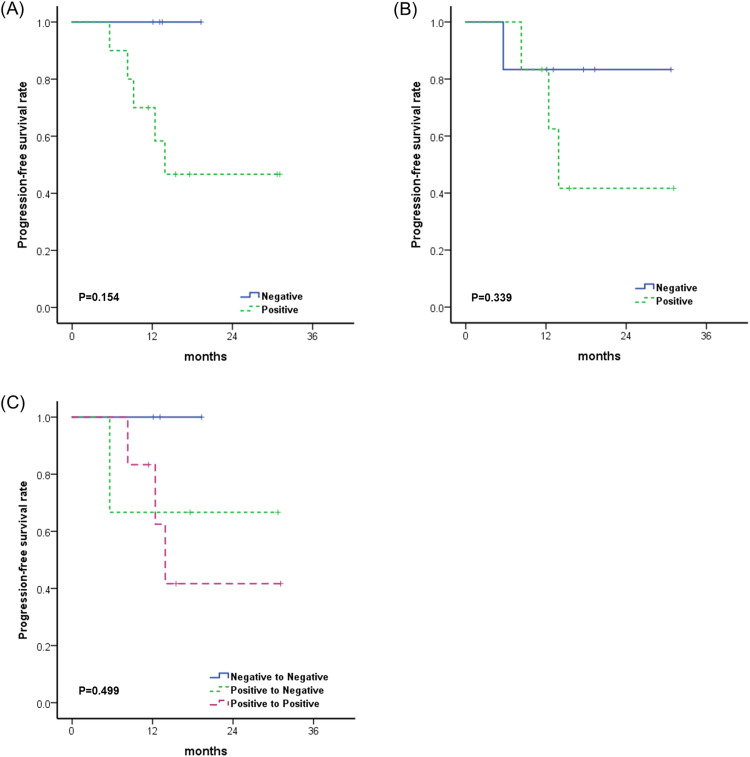
Kaplan-Meir curves for progression-free survival according to preoperative ctDNA positivity (A), postoperative ctDNA positivity (B) and change of ctDNA positivity (C).

After a median follow-up of 17.4 months (range, 11.4–31.1 months), the 1-year and 2-year PFS rates were 78.6% and 58.2%, respectively (Fig 7). The results of univariate analysis of PFS are summarized in Table 6. Among the variables examined, only tumor location showed a significant association with PFS. Extrahepatic tumors exhibited the best prognosis, with a 2-year PFS rate of 100% compared to 0% for intrahepatic bile duct tumors and 53.3% for gallbladder tumors (p = 0.001). Both preoperative and postoperative ctDNA positivity showed trends suggesting an association with PFS; however, the results were not statistically significant (p = 0.154 and p = 0.339, respectively, Fig 8A-B). Preoperatively, none of the 4 patients (0%) with a negative ctDNA result experienced disease progression, whereas 5 out of 10 patients (50%) with a positive ctDNA result showed progression. Postoperatively, 1 out of 6 patients (16.7%) with a negative ctDNA result experienced disease progression, in contrast to 3 out of 6 patients (50%) with a positive ctDNA result. Based on changes in ctDNA positivity status, none of the 3 patients who remained negative experienced disease progression; however, 1 out of 3 patients who showed a change from positive to negative and 3 out of 6 patients who remained positive experienced disease progression (Fig 8C).

**Table 6:** Univariate analysis for progression-free survival.

Variables		n	2-year rate (%)	*p*-value
Gender	Male	8	40.0	0.288
	Female	6	83.3	
Tumor location	Intrahepatic	3	0.0	0.001
	Extrahepatic	6	100.0	
	GB & GB+extrahepatic	5	53.3	
Stage	I – II	9	62.2	0.918
	III – IV	5	60.0	
T stage	T1-T2	12	58.3	0.556
	T3-T4	2	50.0	
Node metastasis	No	10	56.0	0.324
	Yes	4	75.0	
Resection margin	Negative	10	38.9	0.095
	Positive	4	100.0	
Adjuvant treatment	No	9	50.0	0.139
	Yes	5	80.0	
Preoperative ctDNA	Negative	4	100.0	0.154
	Positive	10	46.7	
Postoperative ctDNA	Negative	6	83.7	0.339
	Positive	6	41.7	
ctDNA change	Negative → Negative	3	100.0	0.499
	Positive- → Negative	3	66.7	
	Positive → Positive	6	41.7	

Abbreviations: GB, gallbladder; ctDNA, circulating tumor DNA.

### Longitudinal ctDNA status and tumor markers

**Fig 9:**
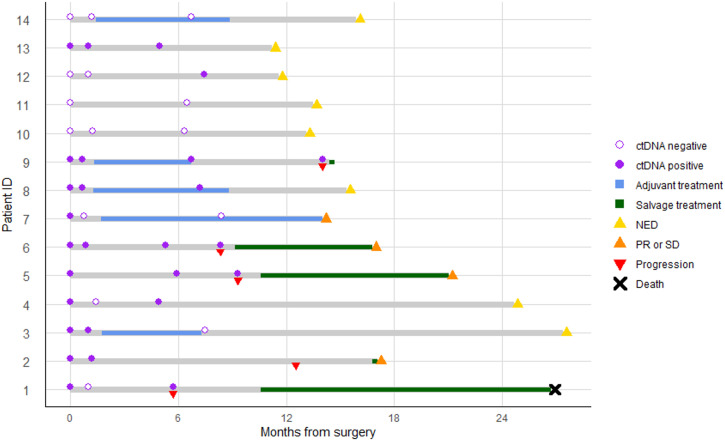
Swimmer plot showing ctDNA status, adjuvant treatment, disease progression and salvage treatment in 14 patients with resected biliary tract cancer. Each horizontal gray bar represents the disease course of an individual patient, with time from surgery (months) shown on the x-axis. Blue and green segments indicate the periods of adjuvant and salvage treatment, respectively. Circles denote ctDNA status at specific time points. Red inverted triangles indicate disease progression, and upright triangles indicate disease status at the last follow-up. Black crosses indicate death. Symbol definitions are shown in the figure.

The longitudinal changes in ctDNA status and serum tumor markers were assessed during follow-up (Fig 9). Among the 14 patients, 5 received adjuvant treatment and 9 did not. To evaluate ctDNA clearance, ctDNA was assessed at 6 months after the start of adjuvant treatment in the treated group, and at 6 months after surgery in the untreated group, along with the timing of recurrence.

Of the 5 patients who received adjuvant treatment, 4 were ctDNA-positive before surgery, and 3 remained positive afterward. Following adjuvant treatment, ctDNA became negative in 1 patient.

Among the 9 patients who did not receive adjuvant treatment, 6 were ctDNA-positive preoperatively. Of these, 5 had ctDNA results available at 6 months postoperatively. One patient showed ctDNA clearance immediately after surgery but became positive again at 6 months, while the remaining 4 patients remained ctDNA-positive throughout. Among the patients who experienced recurrence and had ctDNA samples available at the time of recurrence (n = 4), all showed ctDNA positivity.

Serum tumor markers, CEA and CA19−9 did not demonstrate a consistent correlation with ctDNA status. Among 4 patients with ctDNA positivity at the time of progression, CA19−9 levels were elevated in only 2, and CEA was elevated in just 1 patient (S1 Table).

## Discussion

The findings of this study provide preliminary evidence that tumor-informed ctDNA may serve as a biomarker for detecting MRD in patients with resectable BTC. Both preoperative and postoperative ctDNA positivity demonstrated a correlation with an increased risk of disease progression and poorer PFS, indicating that ctDNA may serve as a sensitive marker for detecting subtle residual disease or potential metastasis following surgical resection. These results suggest that ctDNA could play a vital role in postoperative monitoring, functioning as an adequate guide to adjuvant therapy.

The controversy surrounding adjuvant therapy in BTC highlights the need for more precise methods of patient selection. Although systemic control is critical for BTC, radiotherapy is still important for locoregional control [23]. Therefore, it is crucial to select the right patients for adjuvant therapy to maximize survival outcomes. In this context, ctDNA offers a promising approach for refining the patient selection process. A recent study reported the utility of ctDNA as a prognostic marker and monitoring tool for recurrence in resected extrahepatic BTC undergoing adjuvant chemotherapy [24]. ctDNA may provide improved sensitivity over conventional biomarkers, including CA 19−9 and CEA, for predicting treatment outcomes. In our results, all patients with available ctDNA results at the time of disease progression were ctDNA-positive, while CA19−9 and CEA were elevated in only a small subset (2 and 1 patients, respectively). These findings suggest that ctDNA may be more sensitive than serum tumor markers in detecting recurrence and may have implication for guiding adjuvant therapy decisions, ultimately enabling personalized treatment strategies based on molecular residual disease status. As demonstrated in colorectal cancer and other gastrointestinal malignancies, ctDNA has been found to be highly prognostic in the post-curative resection and adjuvant therapy settings, not only reflecting the presence of minimal distant metastasis but also providing insights into the quality of the surgical procedure itself. Therefore, it is possible that ctDNA may also hold value in BTC, allowing more tailored adjuvant treatment strategies.

Additionally, our findings revealed that the vast majority (99.7%) of somatic variants identified through the WES of tumors were unique to individual patients. This observation is consistent with the result of a recent study on colorectal cancer, which reported that 98.4% of mutations were patient-specific [11]. Consequently, an MRD detection approach with a broad coverage of these patient-specific mutations could also be a sensitive method for identifying MRD in BTC.

This study has several limitations. The small sample size of 14 patients limits the statistical power and precludes drawing reliable statistical conclusions. Therefore, this study should be interpreted as an exploratory investigation. The relatively short follow-up period also prevents the evaluation of long-term outcomes. Furthermore, blood samples were not collected frequently enough during the immediate postoperative period, which could have yielded additional insights into ctDNA dynamics. However, despite these limitations, our findings may indicate a distinct prognosis according to ctDNA positivity, suggesting the possible role of ctDNA MRD as a prognostic marker in BTC. Future studies involving more patients with a longer follow-up are needed to validate our results.

In conclusion, this exploratory study provides preliminary evidence that ctDNA MRD detection may help predict the risk of recurrence and guide adjuvant therapy strategies for patients with resected BTC. However, due to the small sample size and limited statistical power, these findings should be interpreted with caution. Further large-scale studies are warranted to validate the clinical utility of ctDNA in guiding personalized treatment plans and improving clinical outcomes.

## Supporting information

S1 Table Comparison of ctDNA status and serum tumor markers (CA19−9 and CEA) at multiple time points. (DOCX)
