# Supplementary material for: Feasibility of tumor-informed circulating tumor DNA for detecting minimal residual disease in surgically resected biliary tract cancer
Source: PLoS One. 2026 Jan 21;21(1):e0341432. doi: 10.1371/journal.pone.0341432 (PMC12822969; doi:10.1371/journal.pone.0341432)
Supplement: S1 Table — (DOCX) [file pone.0341432.s001.docx]

|  | Before surgery | | | 1 month after surgery | | | 6 months after surgery  or adjuvant treatment | | | At progression | | |
| --- | --- | --- | --- | --- | --- | --- | --- | --- | --- | --- | --- | --- |
| Patient ID | ctDNA | CA19-9 | CEA | ctDNA | CA19-9 | CEA | ctDNA | CA19-9 | CEA | ctDNA | CA19-9 | CEA |
| 1 | **Positive** | Normal | Normal | Negative | Normal | N/A | **Positive** | Normal | N/A | **Positive** | Normal | N/A |
| 2 | **Positive** | Normal | Normal | **Positive** | N/A | N/A | N/A | N/A | N/A | N/A | Normal | N/A |
| 3 | **Positive** | Normal | Normal | **Positive** | Normal | Normal | Negative | Normal | Normal |  |  |  |
| 4 | **Positive** | **Elevated** | Normal | Negative | Normal | Normal | **Positive** | Normal | Normal |  |  |  |
| 5 | **Positive** | **Elevated** | Normal | N/A | Normal | N/A | **Positive** | Normal | N/A | **Positive** | Normal | N/A |
| 6 | **Positive** | **Elevated** | Normal | **Positive** | Normal | N/A | **Positive** | **Elevated** | N/A | **Positive** | **Elevated** | **Elevated** |
| 7 | **Positive** | **Elevated** | N/A | Negative | **Elevated** | Normal | Negative | Normal | Normal |  |  |  |
| 8 | **Positive** | **Elevated** | Normal | **Positive** | Normal | N/A | **Positive** | Normal | Normal |  |  |  |
| 9 | **Positive** | N/A | N/A | **Positive** | **Elevated** | N/A | **Positive** | **Elevated** | Normal | **Positive** | **Elevated** | Normal |
| 10 | Negative | **Elevated** | Normal | Negative | Normal | Normal | Negative | Normal | Normal |  |  |  |
| 11 | Negative | Normal | Normal | N/A | Normal | Normal | Negative | Normal | Normal |  |  |  |
| 12 | Negative | **Elevated** | Normal | Negative | Normal | Normal | **Positive** | Normal | Normal |  |  |  |
| 13 | **Positive** | **Elevated** | Normal | **Positive** | Normal | Normal | **Positive** | Normal | Normal |  |  |  |
| 14 | Negative | Normal | Normal | Negative | Normal | Normal | Negative | Normal | Normal |  |  |  |

S1 Table. Comparison of ctDNA status and serum tumor markers (CA19-9 and CEA) at multiple time points

*Abbreviation* : N/A, not available
